# Outbreak of invasive pneumococcal disease among shipyard workers, Turku, Finland, May to November 2019

**DOI:** 10.2807/1560-7917.ES.2019.24.49.1900681

**Published:** 2019-12-05

**Authors:** Marius Linkevicius, Veronica Cristea, Lotta Siira, Henna Mäkelä, Maija Toropainen, Marjaana Pitkäpaasi, Timothee Dub, Hanna Nohynek, Taneli Puumalainen, Esa Rintala, Merja E. Laaksonen, Thijs Feuth, Juha O. Grönroos, Jutta Peltoniemi, Heikki Frilander, Irmeli Lindström, Jussi Sane

**Affiliations:** 1 Expert Microbiology Unit, Department of Health Security, Finnish Institute for Health and Welfare (THL), Helsinki, Finland; 2 European Programme for Public Health Microbiology Training (EUPHEM), European Centre for Disease Prevention and Control, Stockholm, Sweden; 3 Infectious Disease Control and Vaccinations Unit, Department of Health Security, Finnish Institute for Health and Welfare (THL), Helsinki, Finland; 4 European Programme for Intervention Epidemiology Training (EPIET), European Centre for Disease Prevention and Control, Stockholm, Sweden; 5 Department of Hospital Hygiene and Infection Control, Turku University Hospital (TYKS), Turku, Finland; 6 Division of Medicine, Department of Pulmonary Diseases, Turku University Hospital (TYKS), Turku Finland; 7 Department of Pulmonary Diseases and Clinical Allergology, University of Turku, Turku, Finland; 8 Department of Clinical Microbiology, Turku University Hospital (TYKS), Turku, Finland; 9 Infection Control Unit, Welfare Division, City of Turku, Finland; 10 Finnish Institute of Occupational Health, Helsinki, Finland

**Keywords:** Invasive pneumococcal disease, Outbreak, pneumococcus, shipyard, Finland, Streptococcus pneumoniae

## Abstract

We report an outbreak of invasive pneumococcal disease and pneumococcal pneumonia among shipyard workers, in Turku, Southwest Finland. In total, 31 confirmed and six probable cases were identified between 3 May and 28 November 2019. *Streptococcus pneumoniae* serotypes 12F, 4 and 8 were isolated from blood cultures of 25 cases. Occupational hygiene measures and vaccination of ca 4,000 workers are underway to control the outbreak at the shipyard.

In early October 2019, Turku University Hospital (TYKS) observed several cases of invasive pneumococcal disease (IPD) among shipyard workers. On 3 October, TYKS alerted the Finnish Institute for Health and Welfare (THL) about seven *Streptococcus pneumoniae*-positive blood cultures among pneumonia patients with a link to a shipyard in Turku. Here we describe the undertaken investigations and control measures of the outbreak.

## Description of the outbreak

Of the initial cluster, the first case was admitted to TYKS on 29 August and the last on 3 October. THL had received four *S. pneumoniae* (pneumococcus) blood-culture isolates before the original alert, and serotyped these on 4 October (serotype 4 isolates (n = 3) and serotype 12F isolate (n = 1)).

**Figure 1:**
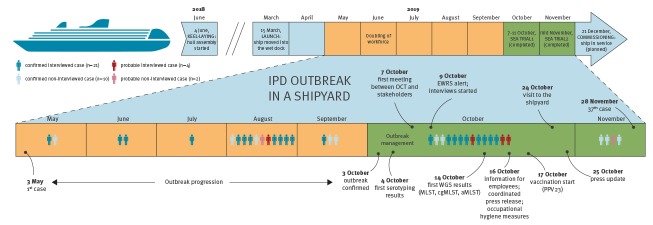
Timeline of invasive pneumococcal disease outbreak at a shipyard, Turku, Southwest Finland, May–November 2019

After confirming an increase in IPD cases among men, between 18 and 67 years of age, in Southwest Finland compared with National Infectious Disease Register (NIDR) baseline data from 2009 to 2018, THL declared an ongoing IPD outbreak at the shipyard. THL formed an outbreak control team (OCT) in collaboration with TYKS, the City of Turku, the Finnish Institute of Occupational Health and the shipyard management to investigate and contain the outbreak (Figure 1).

aMLST: accessory genome multilocus sequence typing; cgMLST: core genome multilocus sequence typing; EWRS: Early Warning and Response System; IPD: invasive pneumococcal disease; MLST: multilocus sequence typing; OCT: outbreak control team; PPV23: pneumococcal polysaccharide vaccine; WGS: whole genome sequencing.

Key events of ship building are marked on the top arrow. Outbreak progression in the months before the alert from Turku University Hospital are coloured in orange, and the months after the alert are coloured in green. Cases are depicted in a chronological order.

## Shipyard workforce

Nearly 7,000 workers access the shipyard area daily. The workforce is multinational: some are employed directly by the shipyard (mainly for hull building), some via subsidiaries and numerous subcontractors (mainly for final stages of ship outfitting).

## Case definition

In order to allow for a broad case finding, a confirmed outbreak case was defined as an individual who had worked at the shipyard after 1 February 2019 and presented with a clinical diagnosis consistent with IPD or pneumococcal pneumonia and had *S. pneumoniae* isolated from blood or cerebrospinal fluid or pneumococcal antigen detected in urine. If there was no laboratory confirmation, the case was defined as probable.

## Epidemiological investigations

We reviewed hospital records and laboratory notifications to the NIDR from TYKS since 1 February 2019 to seek additional outbreak-related cases. As at 28 November, 31 confirmed cases were identified; all except one were men, with cases having a median age of 48 years (range: 19–64). The cases were nationals of Finland (n = 13) or other European Union/European Economic Area (EU/EEA) (n = 16) or non-EU/EEA (n=2) countries. We interviewed both confirmed and probable cases to describe demographics, risk factors for IPD, activities outside work, work tasks and onsite working patterns of cases 10 days before the onset of symptoms. THL issued a communication through the EU Early Warning and Response System on 9 October to inform and facilitate potential case finding across Europe. No cases connected to this outbreak were identified outside Finland.

**Figure 2:**
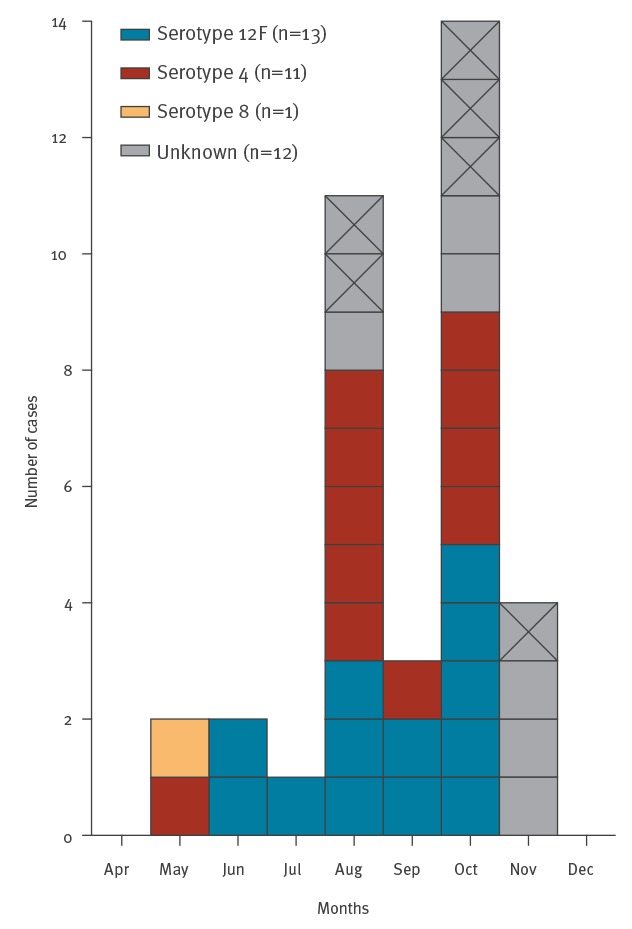
Confirmed and probable cases during an invasive pneumococcal disease outbreak at a shipyard by month of specimen collection and serotype, Turku, Southwest Finland, May–November 2019 (n = 37)

As at 3 December, 30 confirmed cases had been hospitalised and treated for septic pneumococcal pneumonia between 3 May and 3 December, and one case died from pneumococcal meningitis. Additionally, there were six probable cases, all of whom were men of working age and treated at a hospital within the same period (Figure 2). Twenty-five of the 37 confirmed and probable cases were interviewed (Figure 1) and the majority of them were current smokers (n = 19), mostly without underlying conditions (n = 22), working on the final stages of ship outfitting with no common activities outside work (Table). Eight confirmed and three probable cases were identified after the start of the vaccination intervention, with one confirmed case being vaccinated at the shipyard 2 days and another confirmed case vaccinated 1 month before symptom onset. We did not consider the former vaccinated case as a vaccine failure as the immune response had no time to develop, while the laboratory information is pending for the latter one. Additionally, one of three probable cases was also vaccinated 1 month before symptom onset.

Serotypes were not available for six probable cases (marked with a X) and six confirmed cases (four urine antigen positive-confirmed cases without blood culture and two culture-confirmed case).

**Table:** Characteristics of the interviewed cases in an invasive pneumococcal disease outbreak at a shipyard, Turku, Southwest Finland, May–November 2019 (n = 25)

Characteristic	Interviewed cases	
	Confirmed (n = 21)	Probable (n = 4)
**Age (years)**		
Median	50	33
Range	23–64	23–47
**Sex**		
Male	20	4
**Nationality**		
Finnish	11	1
Other EU/EEA	9	2
Non-EU/EEA	1	1
**Underlying medical conditions^a^**		
Yes	2	1
**Health check before/during employment at the shipyard**		
Yes	12	2
**Smoking**		
Yes	15	4
**Occupation and tasks at the shipyard**		
Electrician	7	0
Plumber	3	0
Site supervisor	3	0
Ship builder	2	0
Other interior outfitters^b^	6	4
**Work stage at the shipyard**		
Ship in the wet dock	21	4
**Work environment**		
Indoor	11	4
Outdoor	1	0
Both	9	0
**Exposures^c^**		
Inorganic dust	18	4
Metal fumes	13	2
Solvents	11	3
Other^d^	3	0
**Use of respiratory mask**		
Never	13	2
Occasionally	5	2
Always	3	0

EU: European Union; EEA: European Economic Area.

^a^ Chronic obstructive pulmonary disease, lung cancer or asthma.

^b^ Included one welder/carpenter.

^c^ More than one exposure per case was possible.

^d^ Gases, acids or paint fumes.

Ten confirmed and two probable cases were not interviewed.

## Clinical information

IPD cases presented with X-ray-confirmed pneumonia, with the main reasons for hospitalisation at TYKS being thoracic pain, respiratory failure and high fever. Median duration of hospitalisation was 5.5 days (range: 1–34). Seven confirmed cases required intensive care for respiratory failure. Median stay in intensive care unit was 6 days (range: 1–26). All hospitalised patients rapidly responded to antimicrobial therapy and were discharged to home care.

## Laboratory investigations and whole genome sequencing

Pneumococcal isolates were available for 25 confirmed cases. All isolates were susceptible to benzylpenicillin, clindamycin, erythromycin, tetracycline and vancomycin according to European Committee on Antimicrobial Susceptibility Testing (EUCAST) breakpoints [1]. Serotyping by Quellung identified serotypes 12F (n = 13), 4 (n = 11) and 8 (n = 1) (Figure 2).

**Figure 3:**
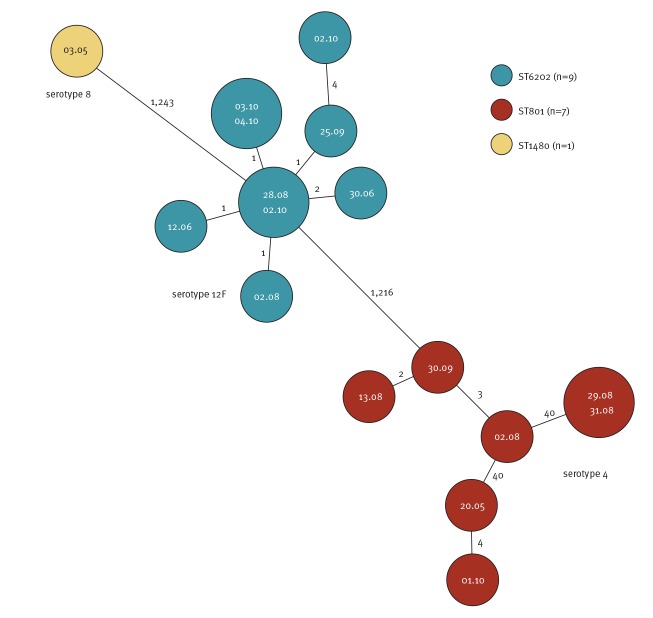
Minimum spanning tree of isolates from cases connected to an invasive pneumococcal disease outbreak at a shipyard, Turku, Southwest Finland, May–October 2019

All outbreak isolates will be whole genome sequenced. At the time of submission, seventeen isolates were analysed by whole genome sequencing to determine the multilocus sequence typing (MLST), core and accessory genome MLST (cgMLST including 1,234 genes; aMLST including 708 genes) profiles. Ridom SeqSphere+ software version 5.1.0 was used to define and analyse the cgMLST and aMLST. The data for this study have been deposited in the European Nucleotide Archive (ENA) at EMBL-EBI under accession number PRJEB35348 (https://www.ebi.ac.uk/ena/data/view/PRJEB35348). Three sequence types (STs) were observed: ST6202 (serotype 12F, n = 9), ST801 (serotype 4, n = 7) and ST1480 (serotype 8, n = 1). Genomes of isolates of the same serotype were similar with ≤ 4 allelic differences within serotype 12F and ≤ 40 allelic differences within serotype 4 (Figure 3). Serotype 4-ST801 was identified in two previous shipyard outbreaks in Vestnes, Norway [2] and Belfast, United Kingdom [3,4]; ST801 has been also reported from IPD isolates in Czech Republic and Portugal [5,6]. ST6202 has been reported from both invasive and non-invasive isolates with several serotypes from China, Russia, Belarus and Singapore [6].

aMLST: accessory genome multilocus sequence typing; cgMLST: core genome multilocus sequence typing; IPD: invasive pneumococcal disease; ST: sequence type.

The date of sampling (day.month) is marked in the centre of each circle, representing an isolate from a confirmed case. Numbers on the branches represent allelic differences between the isolates from cgMLST and aMLST analysis. The number of total investigated targets was 1,424; missing targets in any of the compared isolates were excluded from the analysis.

## Control measures

On 16 October, a decision to implement vaccination and promote hygiene measures to control the outbreak was taken. On the same day, a coordinated press release accompanied by targeted information about IPD and vaccinations on THL’s website was issued in three languages, Finnish, Swedish and English. The shipyard communicated the information to employees by circulating an information bulletin on the vaccine and IPD in three languages, Finnish, English and Russian, in areas accessible to all staff. Simultaneously, the shipyard management implemented hygiene measures and promoted the use of respiratory protective equipment for those exposed to metal fumes and inorganic dust.

Since 17 October, vaccination with pneumococcal polysaccharide vaccine (PPV23), covering all three detected serotypes, and seasonal influenza vaccine have been offered at the shipyard by the shipyard management and the local health authorities. The first target group for vaccination was ca 4,000 employees who worked on the final stage of ship outfitting. As at 7 November, 4,004 workers had received PPV23. Prophylactic antimicrobial treatment of the target group was considered by the OCT, but not implemented, as the potential benefit in preventing further cases effectively and safely was uncertain after consulting external experts.

## Discussion and conclusion

To our knowledge, only two similar IPD outbreaks at shipyards have been previously described: in Norway earlier in 2019 [2], and in Northern Ireland in 2015 [3,4]. Additionally, three IPD cases in workers at a shipyard were reported in Singapore in 2017 [7].

*S. pneumoniae* is a Gram-positive bacterium that can colonise the upper respiratory tract and cause infections such as otitis media, pneumonia and IPD (bacteraemia and meningitis) [8]. Outbreaks of pneumococcal disease occur relatively rarely, but have been described in crowded environments, such as care facilities, garrisons and prisons [9]. Inadequate ventilation in such environments further increases the risk of IPD [10]. In addition to crowding, the level of pre-existing immunity against the infecting strain affects bacterial transmission. Extremes of age, specific underlying co-morbidities, smoking, respiratory viral infections and immunosuppression are known risk factors for IPD [11-13]. Exposure to metal fumes also increases the risk of IPD [14]. In the United Kingdom, it is suggested that vaccination should be considered for employees exposed to welding and metal fumes [15,16]. In Finland, legislation requires employers to offer vaccinations to protect their employees from occupational infectious diseases hazards. However, there are no specific guidelines on offering pneumococcal vaccine to occupational groups exposed to welding and metal fumes.

In Finland, it is mandatory to report IPD to the NIDR, and corresponding isolates are submitted to THL for species verification and serotyping. Since 2010, the childhood vaccination programme includes the 10-valent pneumococcal conjugate vaccine. Pneumococcal conjugate or polysaccharide vaccine use in other age groups is low [17]. In 2018, 760 cases of IPD were reported nationwide (incidence 14/100,000 population), with 5% of cases < 5 years of age and 52% ≥ 65 years of age. Higher incidence is reported among men than women (15 vs 12/100,000 population) [18].

In Turku, as in the Norwegian outbreak [2], both confirmed and probable cases were working in the final stage of ship building in the wet dock, and their work involved interior outfitting. Interviews with cases and discussions with shipyard management revealed that work in the wet dock required the largest workforce compared with previous shipbuilding stages. Interviewees described that many parallel tasks involving exposure to inorganic dust and metal fumes are performed in close proximity. Some of those exposed were not using respiratory masks as these were not required for the main tasks. Workers emphasised that they spent extended working hours in either poorly ventilated or draughty conditions. Furthermore, most cases were heavy smokers (> 11 cigarettes/day, n = 13). In addition to the working conditions, the pneumococcal serotypes identified in this outbreak are associated with a high attack rate [19] and for serotype 12F, a cyclical epidemiologic pattern with outbreaks has been described [20]. We hypothesise that the risk of IPD at the shipyard is likely affected by many of the aforementioned factors, and exposure to different respiratory irritants may have facilitated infection. Extensive vaccination and hygiene measures are underway to control the outbreak. The risk of the shipyard tasks and working conditions will be assessed to identify other preventive strategies. Additionally, retrospective analysis of surveillance data from previous years, as well as further insights into virulence factors and molecular epidemiology, are warranted. Future studies may use other diagnostic criteria to resolve the magnitude of the outbreak.
